# Formulation Strategies to Improve the Stability and Handling of Oral Solid Dosage Forms of Highly Hygroscopic Pharmaceuticals and Nutraceuticals

**DOI:** 10.3390/pharmaceutics14102015

**Published:** 2022-09-22

**Authors:** Liu Han Ng, Jordy Kim Ung Ling, Kunn Hadinoto

**Affiliations:** School of Chemistry, Chemical Engineering and Biotechnology, Nanyang Technological University, Singapore 637459, Singapore

**Keywords:** hygroscopic pharmaceuticals, pharmaceutical coating, nutraceutical encapsulation, cocrystals, solid dosage formulation

## Abstract

Highly hygroscopic pharmaceutical and nutraceutical solids are prone to significant changes in their physicochemical properties due to chemical degradation and/or solid-state transition, resulting in adverse effects on their therapeutic performances and shelf life. Moisture absorption also leads to excessive wetting of the solids, causing their difficult handling during manufacturing. In this review, four formulation strategies that have been employed to tackle hygroscopicity issues in oral solid dosage forms of pharmaceuticals/nutraceuticals were discussed. The four strategies are (1) film coating, (2) encapsulation by spray drying or coacervation, (3) co-processing with excipients, and (4) crystal engineering by co-crystallization. Film coating and encapsulation work by acting as barriers between the hygroscopic active ingredients in the core and the environment, whereas co-processing with excipients works mainly by adding excipients that deflect moisture away from the active ingredients. Co-crystallization works by altering the crystal packing arrangements by introducing stabilizing co-formers. For hygroscopic pharmaceuticals, coating and co-crystallization are the most commonly employed strategies, whereas coating and encapsulation are popular for hygroscopic nutraceuticals (e.g., medicinal herbs, protein hydrolysates). Encapsulation is rarely applied on hygroscopic pharmaceuticals, just as co-crystallization is rarely used for hygroscopic nutraceuticals. Therefore, there is potential for improved hygroscopicity reduction by exploring beyond the traditionally used strategy.

## 1. Introduction

Many active pharmaceutical ingredients (API) and nutraceuticals (e.g., medicinal herbs, prebiotics, dietary fibres) are highly hygroscopic in nature in their solid form [1]. The high hygroscopicity causes multitude of issues such as alterations to their physicochemical properties, which in turn cause difficulties in subsequent downstream formulation processes, instability during their shelf life, and, eventually, adverse effects on their bioavailability [2]. Hygroscopicity, commonly known as ‘moisture-sensitivity’, can be defined as the susceptibility of a solid to absorbing or adsorbing and retaining water from their environment [3]. Highly hygroscopic solids possess polar functional groups or water-attracting binding sites for hydrogen bonding with water, making them relatively hydrophilic or capable of exhibiting ‘water-loving’ properties [4]. By virtue of their hydrophilic properties, water uptake or moisture absorption by the solids can easily occur.

Moisture absorption is typically governed by five factors: (1) difference between the partial vapor pressure of water in the environment and the equilibrium moisture concentration of the solid; (2) surrounding temperature; (3) exposed surface area of the solid; (4) velocity of the moist air; and (5) reactivity of the solid to water, which is a characteristic of the solid itself [5]. All these factors attribute solids’ water-binding tendencies to be driven by moisture gradient and the availability and accessibility of its polar chemical groups such as hydrogen bonding sites to water, which changes depending on the solid’s surface area, chemical composition, crystal structure orientation and presence of any disorganized structure [1,3].

The adverse effects of moisture on pharmaceutical solid dosage forms are threefold. First, it may cause drug degradation through unwanted chemical reactions such as hydrolysis, which forms impurities and reduces the amount of actives in the formulation [2]. Some crystalline bioactives, regardless of whether they are API or nutraceuticals, may transform into hydrates when water is integrated into their lattice in stoichiometric or non-stoichiometric ratios through hydrogen and/or covalent bonding with the anhydrous actives, changing their physicochemical properties, and affecting their solubility, stability, and bioavailability [6]. Poole et al. [7] found that the bioavailability of anhydrate ampicillin was higher than that of trihydrates due to differences in aqueous solubility. Gouda et al. [8] and Ebian et al. [9] discovered that the dissolution rate and bioavailability of nitrofurantoin tablets had reduced after two months under several storage conditions. Otsuka et al. [10] took a step further to study the physicochemical stabilities of nitrofurantoin anhydrate and monohydrate to conclude that crystallographic phase changes in the drug can happen during storage at low or high relative humidity, and that these changes are one of the principal factors in affecting the drug’s bioavailability.

Second, moisture may bring on phase transitions by lowering glass transition temperatures and acting as a plasticizer in amorphous solids [2]. It may induce recrystallization of intended amorphous forms, which were formed for their enhanced solubility, back into stable crystalline forms with decreased solubility and bioavailability. Other than plasticization of amorphous bioactives, it may also cause deliquescence of crystalline bioactives, which is the liquefaction and dissolution of water-soluble solids in the absorbed water [1].

Third, moisture uptake results in the wetting of solids, impacting not only their stability but also powders’ flow property, compactibility, dosing accuracy, and hardness, bringing major downstream formulation and handling challenges [3,11]. Some downstream formulation challenges that hygroscopic or moisture-sensitive solids may face are in processing steps such as powder milling, tablet compounding [12], and powder flowing [13], where the hygroscopic solids may stick to the milling machine and conveyor, leading to dissatisfactory size reduction and stunted flow. In tablet compression, hygroscopic solids may adhere to the punches and cause caking and clumping [12]. In the case of packaging processes such as dry powder filling, hygroscopic solids may cling to the hopper or conveyor and impede the process [12]. Measures such as controlling relative humidity (RH), and the use of adsorbents, lubricants and glidants may be taken in these formulation steps to ensure smoother-sailing processes [14]. Lastly, the final drug form will likely be encased in appropriate packaging to shield them from the effects brought about by the external environment, such as possible chemical and physical degradation from exposure to light, humidity, air, impurities, and mechanical damage. In the case of hygroscopic drug forms, packaging is critical for its protection against moisture in the air, from the time of production to use [14]. Two principle points must be regarded in the packaging of hygroscopic drug forms—(1) it must be conducted under controlled humidity conditions to minimize the amount of water vapor occupying the headspace of the package, and (2) careful consideration must be taken for the choice of the packaging material, to not only ensure that it is inert but that it provides ample moisture protection based on their water vapor permeation rate. Some common packaging materials are polyvinyl chloride (PVC), Aclar (polychlorotrifluoroethylene), and foil [15]. A comprehensive review on the selection of packaging for moisture protection and stability of solid oral drug products has been authored by Waterman and MacDonald, and interested readers may refer to that [16].

Instead of relying on the manipulation of manufacturing and storage conditions to maintain the stability of hygroscopic compounds, pre-emptive actions can be taken in the formulation of bioactives to prevent and/or minimize water absorption for highly hygroscopic active ingredients. By nipping the problem in its bud at earlier stages, it lessens the dependency on the enforcements of tight limitations on conditions such as relative humidity, temperature, and sealing or packaging quality for the rest of the product lifecycle. Furthermore, it may be difficult to maintain controlled processing conditions at all times, as they are prone to uncontrollable variations such as the weather, energy providers, equipment, and operator manoeuvres. In addition, by removing the requirement for such strict controls, it helps to save overall manufacturing cost.

In this review, we present different formulation strategies aimed at improving the stability and handling of oral solid dosage forms of highly hygroscopic pharmaceuticals and nutraceuticals. Examples of such strategies as elaborated later are film coating, encapsulation by spray drying and freeze drying, complex coacervation, co-processing with excipients, and crystal engineering. With regard to nutraceuticals, research based on the potential usage of nutraceuticals have seen exponential growth in the last decade. Furthermore, the global nutraceuticals market is estimated to arrive at USD 578.23 billion by 2025, due to the surge in health concerns and rising popularity of natural therapeutic ingredients. Nutraceuticals cannot replace pharmaceuticals, but can be utilized as a powerful prevention and/or supplementary medicine for some pathological conditions [17]. Therefore, it is important that both pharmaceuticals and nutraceuticals were included in the present review.

The aforementioned formulation strategies (e.g., coating, encapsulation) have been widely employed in solid dosage form formulations of pharmaceuticals for various objectives, e.g., taste masking, controlled release, and improved pharmacokinetics. Numerous review articles discussing the formulation strategies to achieve such objectives have been reported. Interested readers are referred to the review articles by Yang et al. [18], Aguilar-Toala et al. [19], Maderuelo et al. [20], Timilsena et al. [21], Khadka et al. [22], and Varshosaz et al. [23].

On the other hand, to the best of our knowledge, formulation strategies aimed at reducing or controlling the hygroscopicity of pharmaceutical/nutraceutical solid dosage forms has not been reviewed before. Previous review articles on hygroscopicity related issues discussed a single strategy applied mostly on pharmaceuticals, for example, the use of excipients [2], film coating [18,24], and crystal engineering [3] were reviewed. In the present review, for the first time, all the hygroscopicity-reduction strategies that have been pursued for applications in both pharmaceuticals and nutraceuticals’ solid dosage forms were collated, discussed, and compared. The present review focused on latest findings and trends in the field; thus, a large majority of the studies discussed were from 2010 onwards with few exceptions.

It is worth mentioning that the issue of hygroscopicity in pharmaceutical solid dosage form formulation is expected to become bigger in the future because problems with low solubility, poor bioavailability, and polymorphic conversion have continuously plagued the quality of pharmaceutical solid dosage forms [25]. Alternative forms of APIs, such as ionic liquids (IL) and therapeutic deep eutectic solvents (THEDES) have emerged as viable solutions [26]. API-ILs and THEDES have shown great potential in solving the issues of conventional solid dosage forms by providing tailored physicochemical properties such as higher solubility, enhanced thermal stability, faster dissolution rate, and absence of polymorphism [27]. Nevertheless, both of them are known to exhibit hygroscopic properties [28]. The demands to control hygroscopicity of solid dosage forms will only intensify in the near future as these new classes of pharmaceuticals will reach commercialization stage. Therefore, we believe the present review is not only relevant, but also timely.

## 2. Formulation Strategies to Reduce or Control Hygroscopicity

Currently, widely practiced formulation strategies to reduce hygroscopicity of pharmaceuticals or nutraceuticals can be categorized into four groups, i.e., (1) film coating—to form a thin film acting as moisture-barrier around the solid core containing the active ingredients; (2) encapsulation—to envelop the active ingredients with polymers via spray-drying/freeze drying or complex coacervation; (3) co-processing with excipients—to formulate the active ingredients with hydrophobic excipients to divert water away from the actives; and (4) crystal engineering—to transform the crystalline form of the active ingredient to less-hygroscopic crystal forms. Relevant studies on the four strategies were scrutinized, and their findings were discussed in detail.

### 2.1. Film Coating

Forming a moisture-barrier film around the solid dosage core is the most common method to protect hygroscopic cores from contact with water vapor in the surroundings [24]. It presents many advantages, such as fast processing, small space utilization, automation potential, better mechanical film properties, limited increase to tablet size, and ease of customization for specific formulation needs [2]. In general, there are three types of film coating techniques, namely, (1) aqueous solvent coating, (2) organic solvent coating, and (3) dry powder coating.

**Figure 1:**
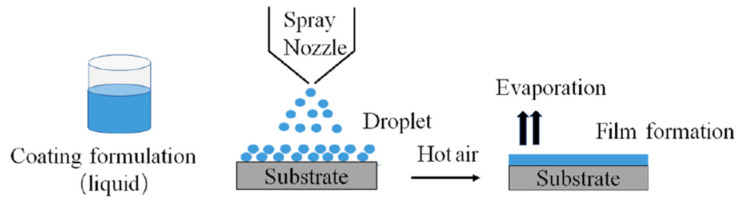
Illustration of film-forming process [24].

For aqueous solvent coating, water-soluble polymers are dissolved to form aqueous coating solutions, whereas for water-insoluble polymers, their micronized particles are dispersed to form aqueous coating suspensions. For organic solvent coating, the polymer is dissolved in organic solvent to form the coating solution. The acquired coating solutions or suspensions are then sprayed onto the dosage cores via atomizing nozzle and subsequent heating process to evaporate the solvents and allow the polymers to fuse into a continuous coating film. A simple schematic illustration describing the coating process is presented as Figure 1.

Solvent coating processes are carried out through either pan coating for larger solid cores such as tablets and capsules, or in fluidized bed for smaller solid cores such as pellets, pills and particles. Although organic solvent coating may outperform aqueous solvent coating in the context of faster operating speed and better uniformity of coating films [24], it is being phased out by aqueous solvent coating due to its limitations such as flammability, explosivity, toxicity, environmental issues, difficulty in the control of residual solvents in the films, and expensive solvent recovery systems. Despite the growing popularity of aqueous solvent coating due to its avoidance of environmental and safety issues as well as high manufacturing expenses incurred by organic solvent coating [2], limitations such as long processing time and high energy usage persist [24]. Furthermore, aqueous solvent coating may not be suitable for extremely moisture-sensitive actives [24].

Therefore, alternative approaches such as dry powder coating have been developed to eliminate or minimize the usage of solvents and water altogether. It works by applying fine polymer particles and additional excipients onto the solid cores’ surfaces, followed by curing at elevated temperatures to form the film coatings. However, this technology is still in its early stages, and more work is required to better it to industrialization [2].

Importantly, the effectiveness of these coating techniques is greatly dependent on the type of polymer used; thus, choosing a suitable coating polymer is necessary. Polymers used as the film coating may be in either crystalline or amorphous form. For amorphous systems, the glass transition temperature (T_g_), which is the temperature at which the polymer transits from glassy to rubbery state, is its main characterizing physicochemical parameter. Amorphous polymers are more susceptible to moisture sorption than crystalline polymers. The absorbed water may act as a plasticizer and lower the T_g_ of the amorphous polymer, inducing the glassy to rubbery state transition, affecting their moisture-barrier property and stability [29].

In general, most of the film-forming polymers for moisture-barriers are synthetic polymers, which can be classified as water-soluble, water-insoluble, and/or entero-soluble polymers which are polymers that do not dissolve in the stomach but in the intestine. Some commonly used water-soluble polymers are polyvinyl alcohol (PVA) and hydroxypropyl methyl cellulose (HPMC). With high solubility in water, these polymers do not affect the drug release and bioactives’ therapeutic properties. However, they have comparatively shorter lifetimes than water-insoluble polymers due to higher susceptibility to degradation by environmental humidity in storage [24].

On the other hand, water-insoluble polymers can alter drug release for sustained or controlled release. Their low water permeability aids them in being moisture barriers. A commonly used water-insoluble polymer is ethyl cellulose (EC). Entero-soluble polymers can deliver moisture protection and enteric functionalities due to being water-insoluble at neutral and acidic pH. Some common examples are shellac and Eudragit^®^ L. Even with unique functionalities, it is important to point out that the solubilities of water-insoluble and entero-soluble polymers may require further enhancements, typically through the addition of excipients or modifications to their thickness to prevent excessive delays to drug release which can hinder therapeutic efficacy [24,29].

Other than polymers, plasticizers and pigments also play important roles in the formulation of film coatings. Plasticizers are used to reduce the T_g_ of films to impart polymer chains with extra flexibility, allowing for the coalescence of molecules and the development of firmer bonds with appropriate tensile strengths. Furthermore, they strengthen the adhesion of the films to the tablet’s surface. Pigments are insoluble additives used to cover intermolecular gaps that have formed during film formation, to form a continuous barrier against moisture [24]. Recent advancements utilizing the mentioned coating techniques are summarized in Table 1 and discussed under the following subsections.

#### 2.1.1. Aqueous Solvent Coating

##### Water-Soluble Polymers

Water-soluble polymers have been widely used for aqueous solvent coating formulation design. The hydrophilicity of water-soluble polymers can help to quickly absorb water to establish water equilibrium with the surroundings, affording stability to solid dosage formulations as a result of reduced susceptibility to moisture. For instance, Zheng, Wu, Hong, Shen, Lin, and Feng [31] used hydrophilic poly(vinylpyrrolidone)’s (PVP) to coat citric acid-containing effervescent tablets in order to overcome its limitations such as sticking and high hygroscopicity. The research team successfully obtained a uniform coating of PVP on the surface of effervescent tablets, providing a physical barrier to lessen direct contact and availability of water for citric acid in the effervescent tablets, from both the surrounding and the PVP layer. The usage of PVP effectively reduced the hygroscopicity and sticking problem, highlighting the efficacy of water-soluble polymers in overcoming the hygroscopicity problems of bioactives.

Aside from PVP, Higuchi, Tanaka, Tamura and Sakata [30] introduced a coating layer comprising PVA and sweetener mannitol to protect the tablets from moisture and to mask their unpleasant taste. From their study, they found that the moisture absorption rate of the PVA/mannitol layer at concentration ratio of 15:2.5–15:4 (*w*/*w*) (smoothest surface) on natural antioxidant L-cysteine was lowest compared with PVA-film-coated and uncoated tablets. They correlated the coating layer’s moisture absorption rate to its surface roughness, where lower surface roughness equates to lower moisture absorption rate.

Another example using PVA-based formulation was shown in the study described by O.Bley, J.Siepmann, and R.Bodmeier [33], where they introduced Opadry AMB^®^ to coat freeze-dried garlic powder, which has many uses such as in medicines for the treatment of infections or the prevention of stroke and arteriosclerosis. The PVA-based formulation was found to be most promising amongst other commercial polymers such as hydroxypropyl methylcellulose (HPMC) and poly(methacrylate-methyl methacrylate) (PMMA) by demonstrating the slowest water uptake when it was in its glassy state. Nevertheless, it is worth noting that upon increasing relative humidity (RH) and storage duration, the moisture uptake rate of PVA-coated tablets increased, which can be attributed to the structural changes in the polymer, specifically of its glassy-to-rubbery state transition. The change in the polymer’s state was confirmed by the change in its glass transition temperature which decreased from 52.1 °C to 17.6 °C after 14 storage days at 75%RH. This finding indicates that the structural changes of polymer coating can strongly affect their moisture-barrier properties, in particular, that a rubbery state of polymers should be avoided since this state is more susceptible to moisture than the glassy state.

The film-coating of a hygroscopic tablet, which was prepared from of a mixture of excipients comprising lactose monohydrate, microcrystalline cellulose, pre-gelatinized starch, magnesium stearate, and colloidal silica, served as another example on how fast absorption of water from the environment can help with hygroscopicity reduction. Eudragit^®^ L 30D-55-coated tablets, which exhibited the most rapid equilibrium, was least hygroscopic compared with other commercial polymers such as Opadry AMB^®^ and Sepifilm™ LP 014 [32].

A combination of polymers can also be used instead of a single polymer. An example is the combination of hydrophilic commercial polymers Eudragit^®^ E PO and Eudragit^®^ RLPO for the coating of moisture-sensitive ranitidine hydrochloride, a treatment for gastroesophageal reflux disease. The coated tablets displayed superior moisture protection over its marketed formulation RANTEC 300, where it gained only 10–15% moisture compared with 35–40% in RANTEC300 after 170 h in 75%RH, proving the effectiveness of hydrophilic polymer combinations as moisture-protective barriers [34].

An interesting point worth highlighting is that aqueous solvent coating may not always be less suitable than organic solvent coating in the film-coating of moisture-sensitive actives. Li, Guo and Heinamaki [35] proved the theory by coating metoprolol tartrate tablets, a hypertension medication, with zein via the aqueous solvent coating and organic solvent coating methods. The aqueous solvent-based zein was found to have lower water vapor permeability than organic solvent-based zein due to its smoother, more densely packed, and smaller colloidal polymeric particles. The water acted as plasticizer of zein to enhance the coalescence of polymer particles, forming more compact film coating.

##### Combination of Water-Soluble and Water-Insoluble Polymers

The moisture-protective benefits of water-soluble and water-insoluble polymers can be reaped simultaneously by using them in combination. Hydrophilic water-soluble polymers can form hydrogen bonds with water and prevent water infiltration into the core, whereas hydrophobic water-insoluble polymers can reduce water permeability due to their lower affinity to moisture. Additionally, their combination can be used to balance moisture protection and drug release profiles. Although this option is attractive, hydrophilic and hydrophobic polymers may not mix uniformly to produce a smooth continuously layer due to their opposing polarities.

By using the coating of calcium chloride as an example, polymeric surface-active agent (PSAA) was added into the mix of hydrophilic hydroxypropyl cellulose (HPC) and lipophilic stearic acid (SA) to aid the solid dispersion of the two opposing phases [36]. It was found that PSAA added at certain concentrations can help to form micelles, allowing tight junctions between the phases, resulting in the formation of a uniform and continuous film. The combination of HPC/SA/PSAA was found to lower water vapor permeability compared with individual polymers, where HPC:SA:PSAA at ratio of 62:25:10 yielded the lowest water vapor transmission rate at 60 g/m^2^ day compared with free HPC film at 180 g/m^2^ day. The combination of polymers also proved to be effective against moisture absorption as HPC/SA/PSAA at the ratio of 62:25:10 exhibited a weight gain of only 3.5% weight compared to 10% in uncoated tablets after 168 h in 75%RH. This suggested that the combination of water-soluble and water-insoluble polymers was feasible in resolving the problem of high water-moisture uptake.

Another example can be seen in the enhancement of moisture-protective ability of hydrophilic pectin by the addition of hydrophobic shellac due to its lower affinity to moisture [47]. Aside from that, the coating of Herniaria glabra L. extract, a plant extract with diuretic and antilithiatic effects, with hydrophilic hydroxypropyl methylcellulose (HPMC) and hydrophobic shellac showed enhanced moisture protection when the weight gain of uncoated tablets decreased from 16.1% to 5.7% in coated tablets at 75%RH, and from 18.2% to 7.5% at 90%RH after around 110 h. This bolsters the claim that a combination of hydrophilic and hydrophobic polymers can be an effective moisture barrier [37].

##### Multi-Layer Coatings

Multi-layer coatings are formed when a core material is coated with more than a single layer of coating around the core. Each layer in a multi-layer coating imparts distinct functions such as taste-masking, impact toughness, moisture-barrier, smoothness, and sustained or controlled release properties. This was found by Ohmori, Ohno, Makino, and Kashihara [38] to be a plausible alternative to sugar coatings to not only overcome its common limitations such as enormous size, high calories, and moisture contents, but provide similar or enhanced functions as effective moisture barriers and taste-maskers. The study experimented on cores that were usually sugar-coated, such as vitamins C, E, B2, calcium pantothenate, and L-cysteine. Instead of sugar coating, a thin sugarless coating was applied. The sugarless coating consisted of several layers that were largely made of HPMC and erythritol as sweetener, namely: an undercoating to halt migration of highly soluble drugs to outer layers; a build-up coating to improve impact toughness; a syrup coating to grant gloss and elegance; and finally, a polishing layer. The thin sugarless coating was found to be less hygroscopic than sugar-coated tablets, proven by how >95% of its drug content remained after 6 months at 40 °C/75%RH compared with <90% in sugar-coated tablets [38].

Another interesting multi-layer coating was formulated by Huang, Tsai, Cheng, Cham, Lai, and Chuo [39] to protect highly hygroscopic pyridostigmine bromide, a drug used for the treatment of neuromuscular disease myasthenia gravis and “Soman” nerve gas poisoning. The multi-layer coating comprised Opadry II as the seal layer to reduce its roughness, Surelease^®^ as the sustained release layer, and Opadry II HP as the waterproof layer. Overall, the multi-layers could reduce the drug’s hygroscopicity significantly, and provided the drug with controlled release properties.

In another study, Min, Park, Hur, Shin, Cho, and Kim [40] developed a multi-layer-coated tablet formulation of choline alfoscerate to control its hygroscopicity. In their study, the choline alfoscerate, used as treatment for the amelioration of cognitive dysfunction in neurodegenerative diseases, was formulated with hydrophobic excipient magnesium aluminometasilicate into granules, and coated with hydrophobic sub-coating Opadry I^®^ and hydrophilic outer coating Opadry AMB^®^. The designed multi-layer coating presented enhanced hygroscopicity over commercially available soft capsule packed Zymax blister film.

#### 2.1.2. Organic Solvent Coating

Although water-insoluble polymer may offer lower water permeability due to its hydrophobic nature, it may not necessarily guarantee better drug stability compared with water-soluble polymers. The coating of aspirin tablets, commonly used for its pain-relieving and anti-inflammatory properties, with hydrophobic shellac displayed notably lower water uptake rates than hydrophilic HPMC-coated tablets, but insignificant difference in the drug’s stability. This was due to HPMC’s ability to bind to water, making the trapped water unavailable for hydrolysis of the drug. However, lower shellac coating levels were needed for similar moisture-barrier properties as HPMC-coated tablets [41].

On the other hand, Reven, Homar, Peternel, Kristl, and Žagar [42] used HPMC phthalate as a coating for solid dispersion of glimepiride and poly(ester amide) hyperbranched polymer, a form of the anti-diabetic oral drug glimepiride with enhanced aqueous solubility and dissolution rate, and managed to reduce its hygroscopicity considerably. Furthermore, hydrogenated rosin was used with hydrophobic plasticizer dibutyl sebacate to coat diclofenac, a nonsteroidal anti-inflammatory drug, resulting in extremely low rates of water vapor transmission compared with shellac-coated drugs due to their highly hydrophobic nature [43].

#### 2.1.3. Dry Powder Coating

##### Polymer Coating

Coating by direct compression outmatches film coating methods in terms of cost-effectiveness, simpler, faster processes, and less need for manpower. It may also be favoured for moisture-sensitive bioactives since it does not use water. External excipients making up the coating are mixed and prepared into granules, followed by manual mixing with the core drug pellets. Finally, they are fed into a die cavity and compressed into tablets using compression machines such as a hydraulic press. For example, pyridostigmine bromide was coated via direct compression with hydrophilic HPMC mixed with water-insoluble excipient Avicel pH 102 to reduce its hygroscopicity [44].

Hot-melt coating is an alternative solvent-free coating technique to prepare dry powder coating, which works by applying polymer powders onto the tablets via a pan-coater followed by melting of the coating layer, or by applying molten polymer directly onto the tablets. Sennae fructus tablets, a herbal laxative, were coated via hot-melt coating with lipids such as Precirol^®^ ATO 5 and Compritol^®^ 888 ATO, followed by aqueous enteric coating Eudragit^®^ L 30D-55, and appeared to have remarkable reduction to their hygroscopicity [45].

##### Sugar Coating

Sugar-coated tablets trump polymer-coated tablets in terms of drug stabilization, ease of swallowing, elegant appearance, and protection against hydrolysis and oxidation of drugs due to sugar crystals having low water permeability and better taste-masking properties and odour [46]. Up till 1950s, sugar coating was the go-to for the pharmaceutical industry [48]. However, since conventional sugar coating requires multi-layers such as sealing, water-proof, sub-coating, smoothing, and syrup-coating layers, it makes the entire coating process time-consuming, complicated, and its productivity and quality highly dependent on operators’ skills [48].

Coupled with problems such as lack of automation, big tablet sizes, high calories, and sugar solution being prone to bacterial growth, it pushed the industry to look towards more efficient coating technologies such as film coating [49]. Although not widely used in the pharmaceutical industry anymore, sugar coating is still commonly employed and growing in trend in the food industry along with global consumers’ preference towards processed, frozen, ready-to-eat, and sugary foods, for the enhancements they bestow to the foods’ appearance, taste, texture, and shelf life [50].

**Figure 2:**
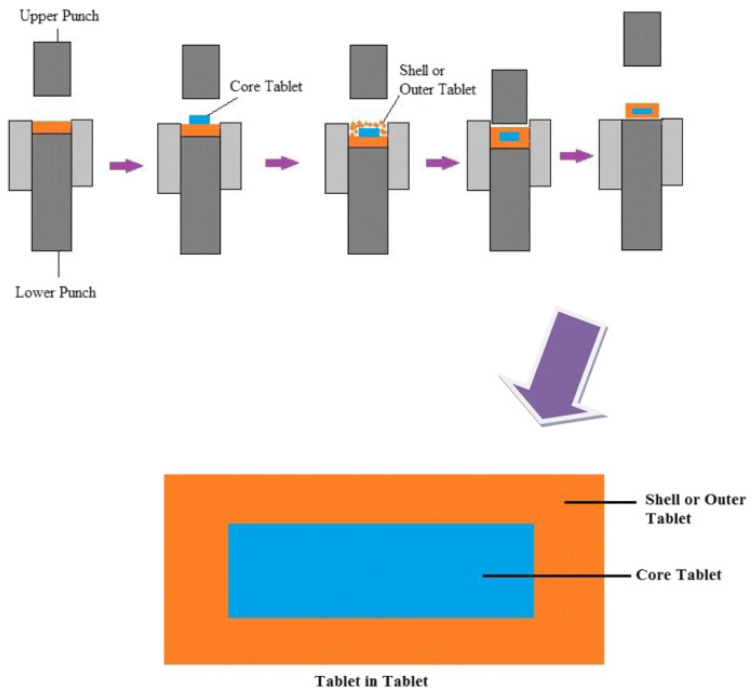
Illustration of direct compression coating process [49].

One of the practices to coat sugar-coating tablets is by using the compression coating technique. A simple schematic representation of the coating process is shown in Figure 2. For example, fructose was coated with amorphous sucrose as outer layer by using one-step dry-coated (OSDRC) tablets manufacturing method via direct compression, which made the whole tablet in a single step without the need to prefabricate the core. An interesting finding was that the amorphous sucrose became crystallized upon compression, blocking water vapor diffusion due to solid crystal formation, gifting it its moisture-barrier properties. OSDRC tablets were found to have greater moisture protection than HPMC tablets [46].

### 2.2. Encapsulation

Encapsulation is the process of coating products with wall materials to shelter them from adverse conditions in the environment. Encapsulation methods are promising techniques for the envelopment of pharmaceutical and nutraceutical bioactives to protect their therapeutic functionalities, control release, mask unpleasant taste, increase solubility, and incorporate them into dry systems. Encapsulation may also help with hygroscopicity reduction for shelf-life extensions and ease of preparation. The encapsulated bioactives in the particle structure is the core, and it is dispersed in a matrix of wall material. Some common encapsulation techniques are spray-drying, freeze-drying, and coacervation [19,51].

The most popular wall materials used for encapsulation include polysaccharides such as maltodextrin (MD), chitosan (CS), gum arabic (GA), and alginate (ALG). Polysaccharides are generally recognised as safe (GRAS) by the Food and Drug Administration (FDA). They have structures that confer stability during controlled release, are cheap, have low viscosities at high ratios, high solubilities in aqueous solutions, good emulsification ability, and are edible and biodegradable. These properties make them fitting for encapsulation techniques. However, their moisture-barrier properties may be limited by their hydrophilicity. Proteins are less popular but used as well, and some common ones are gelatin (GE), whey protein isolate (WPI) and soy protein isolate (SPI). Proteins present advantages such as nutritional benefits, good solubility, emulsification, and gelation abilities, making them suitable for encapsulation techniques too [19,51].

#### 2.2.1. Encapsulation by Spray Drying

**Figure 3:**
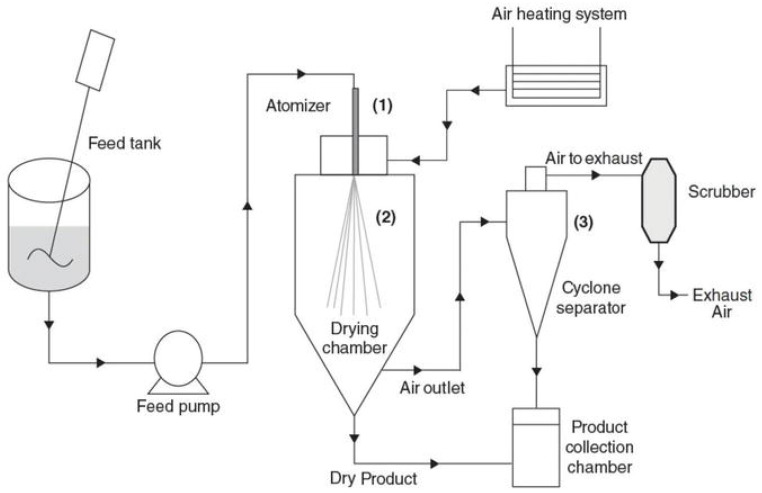
Illustration of spray-drying mechanism (1) atomizer; (2) sprayed droplets; (3) product collector [53].

Spray-drying is the conversion of a liquid solution, emulsion or suspension into dry material via a single step [52]. A simple illustration of the spray-drying process is shown as Figure 3 [53]. Briefly, the dry materials are produced by atomization of the liquid under hot air flow which swiftly removes moisture, creating a solid particulate material that is separated via cyclone and received in a container. The advantages of spray-drying are that they are cost-effective, have short processing time, and can lower the product weight/volume [19,54]. However, due to elevated temperatures during atomization, heat-sensitive materials may be denatured or degraded [55]. Therefore, wall materials such as MD are often used for the protection of these heat-sensitive materials [56].

The recent findings on encapsulation via spray-drying by using single or mixed polymers are tabulated in Table 2, where they are classified either under the ‘single polymer’ or ‘combination of polymers’ group, that are further discussed under this section. An important remark to make is that most of the spray-dried materials presented in this review are nutraceuticals, with most of them being protein hydrolysates. Only one of the materials mentioned, API-IL, classifies as a pharmaceutical.

##### Single Polymer as Wall Materials

Encapsulation of protein hydrolysate core materials with protein wall materials may cause insufficient protection and loss of bioactive properties due to interactions between them as a result of similarity between their compositions [51]. This can be seen in the encapsulation of casein hydrolysate with SPI. Hydrophobic groups of the hydrolysates were hidden due to their interactions with SPI, resulting in conformational changes that exposed more hydrophilic groups to the surface, increasing moisture, water activity and hygroscopicity of the encapsulated hydrolysate [57]. This finding highlighted that protein might not be suitable for the coating of protein cores, due to the possible interactions between the protein core and protein wall which may hinder the moisture-protective abilities of the wall. With this reason, utilization of polysaccharides such as MD as wall material for the encapsulation of protein cores seems to be more plausible.

As can be seen in the recent findings from Table 2, MD is one of the most popular polysaccharide wall materials used for spray-drying. Encapsulation by MD has been shown to decrease hygroscopicity due to its low hygroscopicity and ability to increase T_g_ of spray-dried powders which helps to maintain the powders’ glassy state. These properties contribute to the moisture-protection and stability of spray-dried powders. A few studies had reported MD as a suitable polymer to encapsulate casein hydrolysate, a peptide with physiological benefits and potential anticancer properties. The studies had found that the encapsulated casein hydrolysates had significantly lower hygroscopicity than free hydrolysates due to the increase in T_g_ and low hygroscopicity of the MD film formed around the particles [58,59]. Aside from casein hydrolysate, antioxidant flaxseed protein hydrolysate was also encapsulated with MD in a study by Akbarbaglu, Jafari, Sarabandi, Mohammadi, Heshmati, and Pezeshki [60], where it was found to have pronounced reduction to their hygroscopicity due to being covered by MD film of low hygroscopicity.

An interesting finding is that the molecular weights (M_w_) of wall materials played an important role in controlling the moisture-protective property since it can affect the T_g_. This is shown in the study performed by Y.Z. and H. [62], where the research team used MD with different M_w_ to encapsulate Amaranthus betacyanin pigments. Remarkably, it was found that spray-dried pigments with MD of smaller M_w_ had lower T_g_ and higher hygroscopicity due to the presence of more hydrophilic groups in the shorter chained MD, indicating that wall materials with higher M_w_ are preferred to reduce hygroscopicity. This is confirmed in a later study described by Kurozawa, Park, and Hubinger [61], where chicken meat protein hydrolysate displayed considerable reduction in their hygroscopicity when higher molecular-weighted (M_w_) agents, such as MD and GA, were used.

Moreover, the concentration of MD was found to have a significant effect on the hygroscopicity of spray-dried solids. In the encapsulation of antiviral and anti-inflammatory cactus pear juice with MD, the least hygroscopic powders were achieved with the highest MD concentrations [63]. Likewise, the least hygroscopic powders were obtained with the highest MD concentrations in the encapsulation of antioxidant Acai [64] and antioxidant and antitumor jujube powder [65]. Other than concentrations of core and wall materials, spray-drying parameters such as inlet air temperature and feed flow have been shown to majorly affect the hygroscopicity of spray-dried powders. It was found that in the encapsulation of Acai and jujube powder, lower inlet air temperature and higher feed flow have led to higher moisture content and less hygroscopic powders [64,65]. Overall, the parameters that allow for higher moisture content in the final product have yielded less hygroscopic powders due to minimized affinity for ambient moisture. However, this conclusion may not be generalised for all situations.

Despite the success of MD in the encapsulation of many bioactives, it may not be suitable for the moisture-protection of all bioactives. In the encapsulation of some API-ILs via spray-drying, MD-encapsulated API-ILs rapidly absorbed water and converted from fine powder into very sticky liquid. Instead, when another material such as ethyl cellulose (EC) was used, the encapsulated API-IL remained in powder form in storage. This is probably due to the water-insolubility and immiscibility of EC with API-IL in contrast with the miscibility of MD with API-IL, which allowed EC to protect the API-IL from moisture more effectively than MD [66].

##### Combination of Polymers as Wall Materials

An example of the utilization of a combination of polymers was shown in a study carried out by Yang, Mao, Li, Zhang, Leng, Ren, and Teng [67], where the team combined MD and β-cyclodextrin (β-CD) to encapsulate whey protein hydrolysate. The combination was found to lower the hygroscopicity greatly through the increase in T_g_. The hygroscopicity of MD/β-CD-encapsulated powders at 36.99 g/100 g were lower than MD-encapsulated powders at 43.09 g/100 g as β-CD is more hydrophobic than MD. In another study, a combination of GE and SPI was used to encapsulate casein hydrolysate, and the formulations were found to be less hygroscopic than free hydrolysates. However, variations in GE and SPI concentrations were not seen to cause any appreciable difference in hygroscopicity [68].

Combination of polysaccharides and proteins have also been pursued. Ahmed, Akter and Eun [69] investigated MD and α-amylase usage for the encapsulation of purple sweet potato, a nutraceutical with antimutagenic, anticarcinogenic and antihypertensive properties. MD was used to increase T_g_ to reduce the stickiness of the material, and MD-encapsulated flour was found to have lower hygroscopicity. The presence of α-amylase, which was used to reduce the viscosity of the puree to aid in its extraction from the cell wall matrix, was found to increase hygroscopicity due to its higher moisture, which lowered the T_g_ of the spray-dried flour.

Moreover, a combination of whey protein concentrate (WPC) and sodium alginate (ALG) was used to encapsulate whey protein hydrolysate via spray-drying and freeze-drying. While the hygroscopicity of WPC/ALG-encapsulated powders was not significantly different from the WPC-encapsulated powders, it was found that spray-dried powders had lower hygroscopicity than freeze-dried powders [70]. The superiority of spray-drying over freeze-drying for the encapsulation of bioactives for moisture protection purposes was also shown in the encapsulation of soybean hydrolysates with the combination of SPI and MD. Although the hygroscopicity of both spray-dried and freeze-dried powders were significantly reduced compared to free hydrolysate, spray-drying was still more effective than freeze-drying in that aspect [71].

#### 2.2.2. Encapsulation by Coacervation

**Figure 4:**
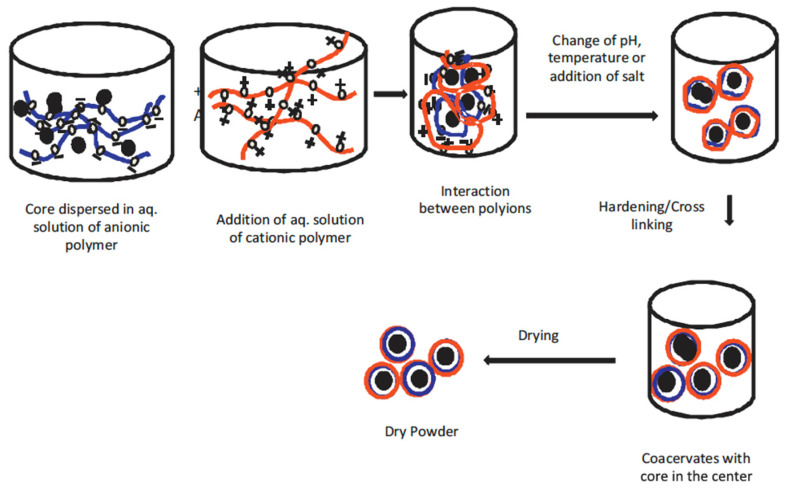
Illustration of complex coacervation process, reprinted with permission from Timilsena, Akanbi, Khalid, Adhikari, and Barrow [21].

Microencapsulation by coacervation is achieved by phase separation of one or more macromolecules from the initial solution followed by envelopment of this phase uniformly around the suspended or emulsified bioactive ingredients in the same media [72]. There are two types of coacervation processes, namely simple coacervation and complex coacervation. Simple coacervation process is when a single polymer is involved and coacervates are formed via a dehydration mechanism through the addition of crosslinker salts or desolvation liquid. A complex coacervation process occurs when two or more oppositely charged polymers form ionic or electrostatic interactions leading to phase separation and formation of coacervates, as shown in Figure 4 [21].

Complex coacervation is more attractive due to its simple processing, lower expenses, scalability, and reproducibility with higher encapsulation efficiency and loading content [21]. It only requires low temperatures for processing, minimizing the evaporation losses of volatile actives or degradation of thermal-sensitive actives. Furthermore, the microcapsules produced via this method are water-insoluble, and has exceptional controlled release and heat-resistance characteristics, making the method attractive [72]. With the growing preference for combined polymers, we focus on reviewing the recent advancement of complex coacervation under this section. In addition, we also included double emulsion complex coacervation for hydrophilic bioactives, as well as a subsection for emulsification and gelation.

Biopolymers used in coacervation encapsulation are often proteins or polysaccharides. The proteins can be either animal-based such as gelatin (GE) and whey proteins, or plant-based such as soy proteins. Widely used polysaccharides consist of alginate (ALG), chitosan (CS), gum arabic (GA), pectin, carragenans, and carboxymethylcellulose (CMC) [21]. Recent findings on the encapsulation by complex coacervation to reduce hygroscopicity are summarized in Table 3. It can be highlighted from the summarized findings that the most popular biopolymer pairs for complex coacervation remained to be GE in combination with other polysaccharides such as GA, ALG, pectin, and CMC.

##### Complex Coacervation

The complex coacervation technique has been applied to numerous active ingredients with the intention to reduce hygroscopicity. It is important to note that the hygroscopicity reduction afforded by the coacervate film is subjected to the water-binding properties of the biopolymers. This depends on the innate quality of the biopolymer, and the extent of intermolecular interactions between the biopolymers which affects the availability of binding sites for ambient moisture. For example, microencapsulation of antioxidant and antitumor capsanthin via complex coacervation of SPI/CS yielded products that exhibited improved stability in low and medium relative humidity. However, it was not as effective in protecting capsanthin against high relative humidity due to the high water-binding capacity of SPI. The incorporation of CS was believed to enhance the moisture-protective property of the SPI/CS film [73].

In a separate study, the freeze-dried antioxidant grape juice extract encapsulated by GE/i-Carrageenan (i-Car) had marked reduction in their hygroscopicity compared with unencapsulated extracts. It was observed that as the amount of GE in GE/i-Car film increased, the water uptake of the extract decreased slightly. This is due to more intermolecular interactions between the biopolymers at higher GE concentrations, resulting in fewer available binding sites for water and a lower water sorption and reduced hygroscopicity [74].

##### Double-Emulsion Complex Coacervation for Hydrophilic Bioactives

**Figure 5:**
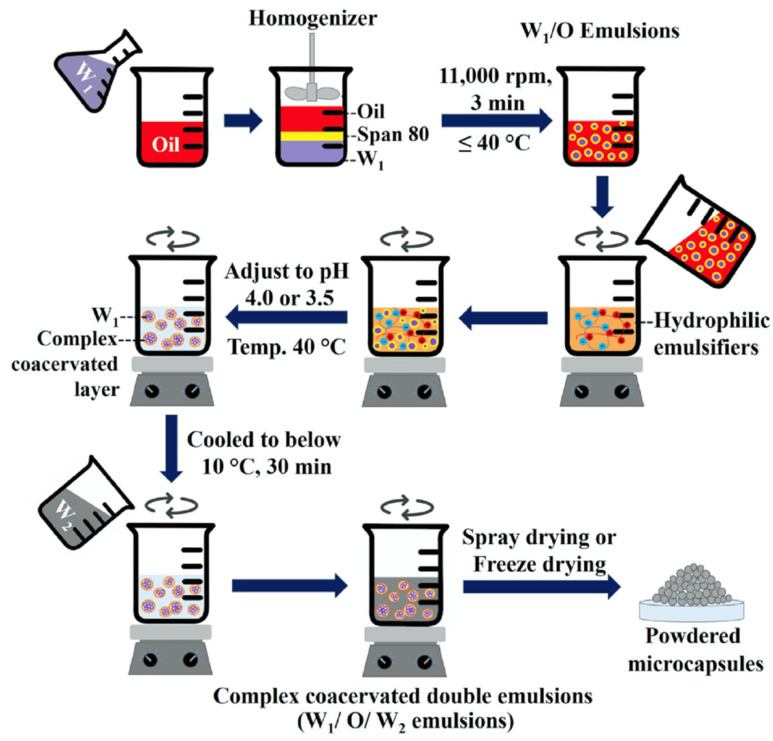
Illustration of an example of double emulsion complex coacervation process, reprinted with permission from Kanha, Regenstein, Surawang, Pitchakarn, and Laokuldilok [78].

Despite being a promising method, complex coacervation is only suitable for hydrophobic bioactives due to the need for phase separation between the core material and the hydrophilic aqueous biopolymer solution. For hydrophilic bioactives, a two-step adaption process is typically required, where they are first emulsified in a primary water/oil (W/O) emulsion, followed by double water/oil/water (W/O/W) emulsion in the biopolymer solution, before encapsulation by coacervation is feasible [78]. A schematic representation showing double-emulsion coacervation is shown in Figure 5.

Hygroscopicity reduction achieved by this method may be attributed to the lower hygroscopicity of the biopolymers used in comparison with the hygroscopic cores. In the encapsulation of aspartame (AS) with GE/GA, the hygroscopicity of the encapsulated AS was not any different from free AS due to the higher hygroscopicity of GE and GA than AS [75]. In contrast, the encapsulation of hygroscopic antioxidant anthocyanin (ANC) with GE/GA afforded it with notably less hygroscopicity than free ANC, where its hygroscopicity reduced from 94.06 g/100 g to 37.05–49.05 g/100 g. This is due to the lower hygroscopicity of GE and GA compared with ANC [76,77]. Therefore, biopolymers used for the encapsulation of hygroscopic cores should be less hygroscopic than the cores for hygroscopicity reduction to be possible.

Other than the hygroscopicity of biopolymers, the drying step after coacervation also affects the hygroscopicity of the products. ANC encapsulated by GE/GA and CS/CMC revealed similar extent in their reduction in hygroscopicity. However, spray-dried extracts were found to have much lower hygroscopicity than the freeze-dried extracts, as there is more driving force and surface area for moisture evaporation in spray drying. Furthermore, freeze-dried samples possess highly porous surfaces, increasing their susceptibility to water absorption [78].

As mentioned earlier, the encapsulation of protein hydrolysate core materials with protein wall materials may result in weakened protection due to the interactions between the core and the wall materials. The hygroscopicity of encapsulation casein hydrolysate with soy protein isolate (SPI)/pectin had been considerably reduced. However, as the amount of content increased, the hygroscopicity increased due to more hydrophobic interactions between the core and SPI, causing the hydrophobic groups of SPI to turn towards the core, decreasing the hydrophobicity on the surface [72]. Therefore, it may be more efficient to utilize other polysaccharides as wall materials for the encapsulation of protein cores. Apart from that, hygroscopic mildronate, a cardioprotective drug, was encapsulated by poly(lactic acid) (PLA) and polystyrene (PS), and had its hygroscopicity significantly reduced in the long run by more than two times, from 66.28% to 26.18% in PLA to 22.04% in PS after 168 h in 75%RH [79].

##### Coacervation by Gelation

Gelation is a form of simple coacervation as it requires a single polymer and a crosslinker to enable coacervation with itself. Although less popular than complex coacervation, a simple coacervation method such as gelation can still be effective to encapsulate hygroscopic bioactives. For instance, different combinations of biopolymers ALG, shellac (LAC) and WPI were used for the encapsulation of probiotic lactobacilli, where crosslinking between ALG or LAC with calcium ions is the main coacervation mechanism for the formation of the films around the cell microcapsules. Films with incorporated LAC demonstrated more obvious reductions in hygroscopicity due to its hydrophobicity and good moisture-protective properties [80].

### 2.3. Co-Processing with Excipients

Solid dosage forms usually comprise the active ingredient coupled with different excipients to aid in its preparation and therapeutic functions. The excipients added should mostly be inert in terms of therapeutic effects, and should not produce undesired alterations such as phase or stability changes in the actives during manufacturing and storage [81]. Excipients may be added to improve the hygroscopicity and stability of solid dosage forms through their interactions with ambient moisture. Excipients can be incorporated into formulations via mixing with or without the help of water, binder, or dissolution solution, followed by melting or solvent evaporation. Examples of common co-processing techniques are wet granulation, dissolution, physical mixing, followed by freeze drying, oven drying, fluid bed drying, or ambient drying. The recent findings on formulation with excipients are tabulated as Table 4.

#### 2.3.1. Amorphous and Low-Crystallinity Excipients

Amorphous excipients can impede on water absorption into bioactives due to their high water affinity and water sorption ability, limiting the amount of available water for binding with actives. This was evidenced by the formulation of antibacterial drug nitrofurantoin anhydrate with several types of excipients—amorphous, partially amorphous and crystalline excipients. The results revealed that only the amorphous excipients, low-substituted hydroxypropyl cellulose (L-HPC) and starch, managed to hamper hydrate formation of the actives at high humidity. The partially amorphous excipient retarded the formation of hydrates at lower humidity, whereas the crystalline excipient did not exert any control over hydrate formation. This was due to the ability of the amorphous excipients in equilibrating slowly and absorbing more moisture over time than the other excipients, conferring more stability to the drug. Starch has high water binding ability due to the presence of plentiful hydroxyl groups and open conformation of glucose monomers [82].

In another study, silicon dioxide as an excipient was added to a formulation of red ginseng extract, which possesses anti-cancer, anti-diabetes, anti-inflammation, and antioxidant properties. The addition of excipient helped to reduce the water sorption rate greatly, improving its stability in storage considerably, converting it from hygroscopic to non-hygroscopic powder. This is due to the amorphous nature of the solid dispersion which provided a bigger surface area for water adsorption, limiting the water absorbed and therefore the recrystallization of the extract [83].

Mihranyan, Stromme, and Ek [84] prepared a formulation of aspirin with excipients of different crystallinity. The lowest degradation rate of the drug was observed in the formulation with the excipient of the lowest crystallinity, low crystallinity cellulose (LCC), although it had the highest moisture. This is possible because these lower crystallinity excipients have more hydroxyl groups for water-binding, which greatly limits the availability and mobility of water to interact with the hygroscopic bioactives.

Similar results were observed in another study performed by Heidarian, Mihranyan, Stromme, and Ek [85], which corroborates the conclusion that the formulation of aspirin with lower crystalline cellulose can reduce its hygroscopicity more than that of higher crystalline celluloses. In addition, the research team deduced that the reason for it is because water is more tightly bonded to LCC than the higher crystalline celluloses, as on average, each water molecule is bonded to LCC by around 3.5 bonds in contrast to around 2 bonds with the higher crystalline celluloses. Since water is more available in higher crystalline celluloses, it is more possible for them to interact with the hygroscopic bioactives, resulting in higher hygroscopicity and chances of hydrolytic drug degradation despite a lower water content.

#### 2.3.2. Hydrophilic Excipients

Similar to amorphous excipients, hydrophilic excipients can confer stability to the bioactives due to their ability to absorb substantial amounts of water and bind to them tightly, restricting the availability and mobility of water to reach the hygroscopic actives. For example, it is known that most of the time, wet granulation of Traditional Chinese Medicine (TCM) will fail to produce granules and instead yield muddy products due to the high hygroscopicity of the extracts. For that, porous excipients such as calcium silicate (FLR) were added to the formulation containing TCM, as described in the study reported by Hirai, Ishikawa, and Takahashi [86]. The addition of FLR allowed the formation of granules due to the high water-sorption ability of FLR, where it can carry up to 4 to 5 times its own weight of water due to its abundance of pores, transferring water very slowly to the extract, successfully yielding granules.

Although hydrophilic polymers can absorb moisture to produce a formulation with a high water content, it does not equate to increased hygroscopicity or destabilization of the bioactives. This is evidenced in the study carried out by Moribe, Sekiya, Fujito, Yamamoto, Higashi, Yokohama, Tozuka, and Yamamoto [87], where they prepared formulation of limaprost, a vasodilator with antithrombotic effect, with polymers such as dextran 40, dextrin, and pullulan. Even though the water contents after storage were very high, the excipients managed to limit water mobility and prevent moisture from interacting with the active, producing stable products.

The amount of excipient used has a big impact on product hygroscopicity. As the ratio of excipients used increase, the collective water-binding ability of excipients increase, limiting more water from having access to the bioactives. This can be seen in the formulation of several herbs with dextran. As the mass of dextran increases, the number of accessible water binding sites increase, decreasing the hygroscopicity of the formulation by dilution, as water binds to both the extract and dextran equally [88].

A similar trend was also observed in the formulation of physalis peruviana fruit extract, a traditional medicine used for treatment and prevention of pterygia, diabetes, and tooth decay. Bernal et al. (2016) formulated the extract with a combination of corn starch and microcrystalline cellulose, where the addition of large proportions of the excipients in comparison to the amount of extract changed the powder from moderately hygroscopic to slightly hygroscopic, preventing deliquescence as a result of the high proportion of water-absorbent excipients [89].

#### 2.3.3. High T_g_ Excipients

As mentioned earlier, maltodextrin (MD) has the ability to raise glass transition temperatures (T_g_) of spray-dried powders to maintain their glassy states, contributing to their moisture-protection and stability. MD can exhibit similar properties as an excipient for amorphous bioactives. For example, Sablani, Shrestha, and Bhandari [90] formulated raw data with MD, which saw the reduction in its hygroscopicity, suggesting that a high molecular weighted (M_w_) polymer such as MD can be used as a potential excipient to raise the T_g_ and reduce the hygroscopicity, stickiness, and improve stability. This was similarly seen in the formulation of several herbs with dextrans. The addition of high M_w_ dextrans helped to raise the extracts’ T_g_ which counteracted the plasticizing effects of moisture and decreased the molecular mobility of the extracts, reducing their hygroscopicity and tackiness [88].

#### 2.3.4. Non-Hygroscopic and Inclusion Complex-Forming Excipients

As illustrated in the previous sections, most of the recent studies focused on using hydrophilic or water-binding excipients to bind to water in order to prevent them from interacting with hygroscopic bioactives. However, non-hygroscopic excipients can also be used to reduce hygroscopicity due to their water-repellent properties. For instance, the formulation of highly hygroscopic anti-vertigo drug betahistine dihydrochloride with non-hygroscopic excipient Quick Tab™ exhibited a lack of moisture uptake and pronounced enhancement to their stability due to the presence of non-hygroscopic tricalcium phosphate in the excipient [91]. Perhaps more studies can be carried out on other non-hygroscopic excipients to highlight their potential as hygroscopicity-reducing additions to formulations.

Other than water-binding or repelling excipients, another league of excipient which reduces hygroscopicity due to their interaction with hygroscopic bioactives was found in study conducted by Maeda, Iga, and Nakayama [92]. The research team formulated the highly hygroscopic betahistine with β-cyclodextrins (β-CD) to prevent its rapid deliquescence and significantly improved its stability. This is due to the hydrophobic cavity in β-CD, which formed inclusion complexes with the hydrophobic molecules of betahistine, stabilizing it. Though uncommon, this provides an interesting doorway to discover more inclusion-complex-forming excipients which can widen the selection pool of hygroscopicity-reducing excipients.

### 2.4. Crystal Engineering

**Figure 6:**
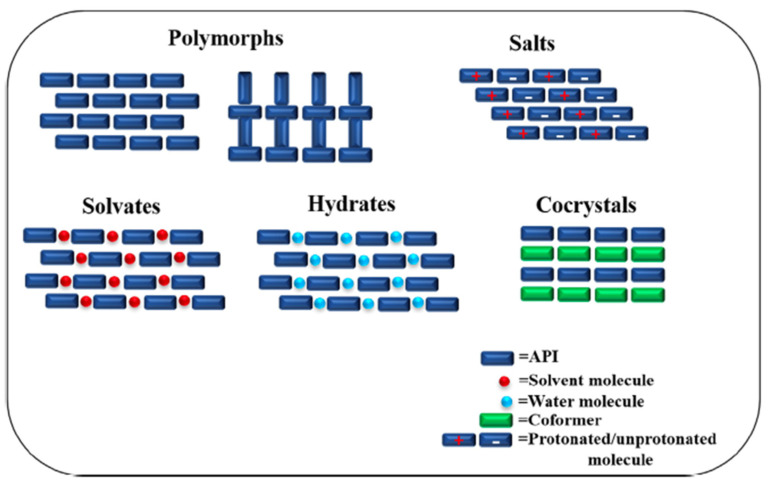
Illustration of different forms of API [93].

Since the structure of a solid affects its properties, the physical stability of bioactives may be boosted by alteration to its crystal packing arrangements via crystal engineering for the formation of different crystals such as salts, polymorphs, hydrates, solvates and cocrystals, as illustrated in Figure 6. However, some of these crystal structures may encounter limitations. For example, only molecules with ionizable groups can form salts, or that hydrates and solvates are not stable due to the susceptibility of water or solvents to be lost from the structure over time. In contrast, many active ingredients may form cocrystals with an appropriate co-former. Therefore, in recent years, cocrystals have been observed to be one of the most popular methods utilized for the improvement of physicochemical properties of active ingredients, through the modification of the crystal structure without changes to its therapeutic functions. Cocrystals are multicomponent systems consisting of two or more individual components of active ingredients and co-formers in a stoichiometric ratio, bonded via non-ionic and non-covalent interactions such as hydrogen bonds in a crystal lattice [93].

Many studies on the formation of cocrystals have been reported, and recent studies related to hygroscopicity control are summarized in Table 5. The co-crystallization of pharmaceuticals to control hygroscopicity has been reviewed by Thakur and Thakuria [3] published in 2020. Their review offered a few reasons for stability enhancements by cocrystals. They mentioned that the aqueous solubility of the co-former is paramount, since even a tiny amount of highly water-soluble co-former can drastically impact its hygroscopicity. Another factor that was hypothesized for better physical stability was efficient crystal packing, although there were also studies that agreed otherwise. The review also mentioned that a discrepancy in the number of hydrogen bond acceptors and donors in a compound may lead to hydrogen bonding unsaturation in a crystal, leading to hydrate formation. This point appeared to be credible with numerous studies coming to the same conclusion. This knowledge is in line with the present review as it is the one of the key reasons mentioned for hygroscopicity improvements from the gathered studies in this section. Other interesting points mentioned in Thakur and Thakuria [3] were that the inclination for hydrates formation may be due to the exposure of hydrophilic functional groups as a result of surface anisotropy, or due to crystal defect sites generated during processing. Since most of the studies published before 2019 were covered in Thakur and Thakuria [3], the present review focuses on discussing the cocrystals studies for hygroscopicity control reported after 2019.

The formation of cocrystals has demonstrated that the reduction in hygroscopicity was a result of the formation of hydrogen bonds between the cocrystal compounds which reduced the availability of hydrogen-bonding sites available for interactions with water. In addition, some studies highlighted hygroscopicity reduction as a result of crystal packing arrangements, where the hygroscopic bioactives were packed away from exposure to ambient moisture. The preparation of cocrystals is widespread and mainly divided into solution-based techniques such as solvent evaporation, antisolvent method, cooling crystallization, reaction co-crystallization, and slurry conversion, and solid-based techniques such as neat grinding, liquid-assisted grinding, and melting crystallization [93].

#### 2.4.1. Co-Crystallization by Solvent Evaporation

Solvent evaporation is the most used method in the preparation of cocrystals. It works by dissolving the cocrystal components completely in an appropriate solvent at stoichiometric ratio, followed by evaporation of the solvent [93]. The formation of cocrystals was shown to reduce the moisture absorption. For instance, the cocrystals of the myotropic drug phloroglucinol with co-former natural steroid hormone progesterone displayed notable reduction in moisture absorption, transforming into a non-hygroscopic compound [94]. On the other hand, Berberine chloride (BCl), a treatment for diarrhoea, abdominal pain and gastroenteritis, was paired with several aliphatic dicarboxylic acid co-formers to form cocrystals that adsorbed negligible moisture, with some resisting the transformation to hydrates at high relative humidity [95].

The behaviour of co-crystallization was also demonstrated in the study performed by Watanabe, Ito, Suzuki, Terada, and Noguchi [96]. They found that the cocrystals of isosorbide, a potent hypertonic agent, with co-formers piperazine, hydrochlorothiazide, 3,5-dihydroxybenzoic acid and gallic acid, had demonstrated higher critical relative humidity and reduced deliquescence, suggesting that the formation of stable solid formulations out of hygroscopic drugs is possible via the cocrystal route. In addition, palmatine chloride, which has antidepressant, pain-relieving, sedative, anti-inflammatory, antifungal, and antibacterial properties, was co-crystallized with co-former gallic acid (GaA). The resultant cocrystals exhibited considerable reduction in hygroscopicity, as GA molecule and methanol inserted themselves tightly into binding sites between chloride anions and ambient water molecules during the self-assembly of cocrystals, hence decreasing the binding ability of the cocrystal with environmental moisture [97]

Sun, Jia, Wang, Liu, Li, Han, and Gong [98] also found that co-crystallization of the anti-diabetic drugs metformin (MET) and epalrestat (EP) showed marked reduction in hygroscopicity, and slightly better hygroscopicity over commercially available metformin chloride (METCl). This is due to the crystal packing formation, where MET cation was positioned in the middle and wrapped by EP anions in EP-MET cocrystal. EP anions acted as a physical barrier to prevent water from contacting the hydrophilic MET. Secondly, the number of potential hydrogen bonding sites in EP-MET is lower than METCl and MET, reducing the availability of water-binding sites.

#### 2.4.2. Co-Crystallization by Liquid-Assisted Grinding

Liquid-assisted grinding uses the addition of a tiny amount of liquid on top of manual or mechanical grinding to yield cocrystals [93]. Berberine chloride (BCl) formed cocrystals with co-former L-Lactic acid, significantly reducing moisture sorption which prevented its transformation into hydrates and allowed it to be stable at high humidity [101]. Another cocrystal of BCl formed with co-former citric acid (CA) also considerably reduced moisture sorption and prevented hydrate transformation, exhibiting greater physical stability. The stabilization of BCl was granted by the dense hydrogen bonding network comprised of strong interactions between Cl^−^ anions of BCl with two carboxylic groups in two neighbouring CA molecules, which must be overcome by water molecules for hydration to happen. Due to the high energetic barrier, the hygroscopicity of BCl-CA was reduced significantly [102].

#### 2.4.3. Co-Crystallization by Solvent Evaporation and Liquid-Assisted Grinding

Studies presented in this section used solvent evaporation and liquid-assisted grinding with different co-formers. Cocrystals of antiepileptic drug sodium valproate with co-formers carbamazepine and tromethamine had pronounced reduction in water absorption at high humidity over a week [99]. Cocrystals of amorphous vemurafenib (VEM), a drug used to treat metastatic melanoma and Erdheim–Chester disease, with different camphorsulfonic acids presented improvements to its hygroscopicity due to the reduction in available hydrophilic binding sites on VEM for water as they were occupied by the intermolecular bonds between VEM and the co-formers [100].

#### 2.4.4. Co-Crystallization by Neat Grinding

Neat grinding method calls for energy input via manual grinding such as mortar and pestle, or mechanical milling, with no requirements for solvent [93]. Cocrystal of antibacterial agent levofloxacin (LVFX) with co-former metacetamol (AMAP) was non-hygroscopic. It resisted transformation into hydrates due to the unavailability of LVFX nitrogen atom for water-binding as it was linked to the hydroxyl group of AMAP via hydrogen bonding [103].

#### 2.4.5. Co-Crystallization by Melt Crystallization

Melt crystallization is carried out by melting the components of the cocrystals completely, then cooling the melt to different temperatures at various timepoints to allow for the growth of the cocrystals [104]. L-lactic acid cocrystals were attained with co-formers D-tryptophan and 3-nitrobenzamide, which allowed the acid to stay stable and not deliquesce [104].

## 3. Conclusions and Perspectives

The present review showed that similar strategies to those employed in addressing low aqueous solubility (e.g., coating, encapsulation, co-processing with excipient co-crystallization) have been pursued to reduce hygroscopicity of solid dosage forms of highly hygroscopic pharmaceuticals/nutraceuticals. Film coating represents the most established and popular method for hygroscopicity control in solid dosage form of pharmaceuticals and nutraceuticals. Water-soluble and/or water-insoluble polymers have been utilized in film coating to form moisture-barrier films surrounding API or nutraceutical particles. Water-soluble polymers can rapidly attain water equilibrium with the surrounding, reducing the actives’ susceptibility to ambient moisture. Water-insoluble polymers deflect moisture due to their hydrophobic natures. Aqueous solvent coating and dry powder coating techniques should continue to replace and gradually phase out organic solvent coating due to the latter’s environmental and safety issues. The present review showed dry-powder coating techniques are not as widely employed as the liquid-based coating techniques. Greater emphasis should be placed on these non-solvent coating techniques due to the advantages they offer, such as shorter processing times, lower energy usages and suitability for moisture-sensitive actives.

Besides film coating, encapsulation by either spray-drying or coacervation has emerged as another widely studied method for hygroscopicity control. Coacervation encapsulation, nevertheless, is not as widely employed as spray drying encapsulation. In spray drying, one of the most popular polymers is MD due to its low hygroscopicity and ability to increase the levels of T_g_ of spray-dried powders. However, MD may not be suitable for all types of active ingredients, for example, in the case of API-ILs where their miscibility allowed water to permeate and reach the core. More studies should be carried out on other types of polymers, perhaps with low hygroscopicity and high M_w_ as well, to expand the list of suitable polymers for the spray-drying of hygroscopic actives. Spray drying parameters that allowed for higher moisture content in the final product had yielded less hygroscopic powders due to minimized affinity for moisture in the environment, but this may not be generalised for all scenarios. Although spray-drying is more effective than freeze-drying in reducing the hygroscopicity of powders, freeze-drying may be explored for more active ingredients, especially heat-sensitive products that are not suitable for spray-drying. Thus far, the application of spray-drying for hygroscopicity control, nevertheless, was largely limited to the encapsulation of nutraceuticals. Being highly effective, spray drying encapsulation should be utilized in the future for controlling hygroscopicity in pharmaceuticals as well.

One drawback observed in the encapsulation strategy by either spray-drying or coacervation is that although most of the bioactives encapsulated via these methods had reduced hygroscopicity, some of the encapsulated cores such as hydrolysates, pigments and anthocyanin, still exhibited rather high hygroscopicity. The high hygroscopicty was perhaps due to the hydrophilicity of the cores or hydrophilicity of the wall materials that had led to their limitations as moisture-barriers. For coacervation encapsulation, other combinations of biopolymers, especially those with extensive intermolecular interactions between them to minimize binding sites for water, and those with high hydrophobicity or lower hygroscopicity, may be studied. For both of the encapsulation methods, encapsulation of protein cores may not be suitable with protein walls due to possible interactions which may obstruct the moisture-protective ability of the protein wall. Therefore, it may be preferable to apply polysaccharide walls for protein cores.

Besides protecting the bioactive cores from moisture by film coating or encapsulation, the third strategy for hygroscopicity control is by co-processing with excipients. The mechanisms of the reduction in hygroscopicity in co-processing with excipients are different depending on the types of excipients used. Water-binding excipients act as absorbents to bind to water tightly and prevent them from contacting the bioactives, high T_g_ excipients help to maintain the powders’ glassy states, non-hygroscopic excipient act as repellents of moisture, and inclusion-complex-forming excipients act as shields for the bioactives. Since there are limited studies conducted on non-hygroscopic and inclusion-complex-forming excipients, they can be focused upon to expand the list of hygroscopicity-reducing excipients.

The fourth strategy for hygroscopicity control is by crystal engineering, whereby the crystal packing arrangements of bioactives is altered to modify their physical stabilities. The present review has focused on cocrystals, but there may be other crystal forms that may benefit hygroscopicity reduction but were not explored in this paper. Out of all the crystal forms, cocrystals emerge as an overwhelmingly popular method. Cocrystals reduce hygroscopicity by occupying their water binding sites with hydrogen bonds between the cocrystal compounds, or by tucking the hygroscopic actives away from exposure to the environment. Nevertheless, co-crystallization to control hygroscopicity has predominantly been applied on pharmaceuticals. Being similar in molecular weights, co-crystallization should be explored for small-molecule hygroscopic nutraceuticals (e.g., flavonoids). At its current state of development, co-crystallization, however, remains a highly empirical process, particularly in the selection of optimal co-formers; thus, its applications for hygroscopicity control can be more technically challenging at the conceptual design stage than the other three strategies.

In the future, a combination of the four strategies can be explored to improve control of hygroscopicity and enhance stability of the bioactives. For example, the hygroscopic bioactive core can be co-processed with moisture-repellent excipient, followed by film coating to yield a superior formulation compared with that prepared by a single strategy. Lastly, despite the significant impacts of high hygroscopicity in pharmaceutical/nutraceutical solid dosage formulation, the present review showed that based on the number of works available in the literature, research efforts on hygroscopicity control in solid dosage formulation remain lagged behind compared with the efforts put in to address other important formulation issues, such as low aqueous solubility, low permeability, and poor tabletability.

## Figures and Tables

**Table 1:** Film coating techniques to reduce hygroscopicity.

API/ Nutraceuticals	Coating Technique	Film/ Coating Material	Key Findings	Ref.
**Aqueous solvent coating**				
L-cysteine	Pan-coating	Sugarless layer: Polyvinyl Alcohol (PVA) and mannitol (sweetener)	At 25 °C/75%RH, moisture absorption rates increased in this order: uncoated tablets (~7%) > PVA-film-coated tablet > sugarless layer-coated tablets (0.25%). It was lowest at mannitol:PVA concentration of 15:2.5 and 15:4 (*w*/*w*).	[30]
Citric acid/Sodium bicarbonate effervescent tablets	Fluid bed	Poly(vinylpyrrolidone) (PVP)	Moisture absorption rate constants (within 15 min) of coated tablets (0.34–0.50) was lower than uncoated granules (0.67) and commercial vitamin C ET (0.71), showing decreased hygroscopicity.	[31]
Lactose monohydrate, microcrystalline cellulose, pre-gelatinized starch, magnesium stearate and colloidal silica	Fluid bed	Eudragit^®^ L 30D-55 PVA-based Opadry AMB^®^ HPMC-based Sepifilm™ LP 014	Sepifilm and Opadry films were more hygroscopic than Eudragit film. Eudragit film was the most effective coating for limiting moisture sorption.	[32]
Freeze-dried garlic powder	Pan-coating	Amorphous polymers: HPMC-based Methocel^®^ E5 Eudragit^®^ E PO Presence of crystalline in polymer: PVA-based Opadry AMB^®^ (Anti-plasticizing agent: PVP)	Opadry AMB^®^ was chosen due to its most promising moisture-protective ability at its glassy state. The addition of anti-plasticizing agent increased water uptake instead, and was deemed unsuitable to enhance the polymer’s performance.	[33]
Ranitidine Hydrochloride	Pan-coating	Eudragit^®^ E PO and Eudragit^®^ RLPO	Eudragit RLPO 10% and Eudragit E PO 10% coated tablets absorbed less moisture at all the tested conditions. In 350 h at 75%RH, it absorbed 15–20% compared with uncoated tablets at 45–50%. In 200 h at room temperature (RT), it absorbed 4–6% moisture compared with uncoated tablets at 8–10%. In 170 h at 75%RH, it absorbed 10–15% compared with marketed formulation RANTEC 300 at 35–40%.	[34]
Metoprolol tartrate tablets	Pan-coating	Water-insoluble Zein Plasticizer: PEG400 or glycerol	Water vapor permeability of aqueous-based coating was lower than organic-based with PEG400.	[35]
Calcium chloride tablets	Fluid bed	Lipophilic substance: Stearic acid (SA) Water-soluble film forming polymer: Hydroxypropyl cellulose (HPC) Polymeric surface-active agent (PSAA)	This combination of polymers helped to lower water permeability of the film compared to individual component films, where HPC:SA:PSAA at ratio of 62:25:10 had the lowest water vapor transmission rate at 60 g/m^2^ day. The uncoated tablets had the highest moisture absorption through the test period compared to coated tablets. HPC:SA:PSAA at ratio of 62:25:10 had the highest sealing capability, with a weight gain of only 3.5% in RT/75%RH after 168 h compared with 10% for uncoated tablets.	[36]
Herniara glabra L. extract	Fluid bed	Lipophilic/enteric substance: Shellac Water-soluble film forming polymer: Hydroxypropyl methyl cellulose (HPMC) Plasticizer: Stearic acid (SA), PEG1500, PEG400 Pigment: Titanium dioxide	Combination formulation of 25% HPMC, 20% shellac, 10% PEG 1500, 29.6% PEG400, 5% tween80, 10% titanium oxide and 0.4% acid red 2 offered good protection against moisture compared with the core tablets, with its weight gain decreasing from 16.1% in core tablets to 5.7% at 75%RH, and from 18.2% to 7.5% at 90%RH after around 110 h. The long-term and accelerated stability studies showed stability of formulation.	[37]
Vitamin C, E, B2, calcium pantothenate, L-cysteine	Pan-coating	Sugarless layer: Undercoating (UC) 2%: HPMC 10%, Purified Water (PW) 90% Build-up coating (BC) 38%: Erythritol 18.2–22%, talc 10.6%, TiO_2_ 0.8%, MCC 0–3.8%, powdered acacia 4.6%, PW 62% Syrup coating (SC) 5%: Erythritol 34.2%, PEG6000 3.8%, PW 62% Polishing: Mixture of waxes, Carnauba wax and white beeswax	Lower hygroscopicity of the sugarless coated tablets was confirmed by the stability of the actives after storage at 40 °C/75%RH for 6 months under closed condition, where >95% drug content remained compared to <90% in sugar-coated tablets. Stability of the actives after storage at 40 °C/75%RH for 1 month under open conditions left >95% drug content in sugarless coated tablets, which was higher than uncoated tablets with <85% drug remaining. The stability and therefore hygroscopicity of sugarless coated tablets were superior to uncoated tablets, and similar to sugar-coated tablets.	[38]
Pyridostigmine Bromide	Fluid bed	Seal layer coating: Opadry II Sustained release layer coating: Surelease^®^ Waterproof layer coating: Opadry II HP	At 25 °C/60%RH, 30 °C/65%RH and 40 °C/75%RH, the uncoated core pellets had their moisture absorption potential increased after 4 h, in contrast to the insignificant increase in moisture absorption of coated pellets up to 4 weeks. Coated pellets’ hygroscopicity was significantly less compared to pure PB which rapidly transformed from solid to liquid state within 10 min.	[39]
Choline Alfoscerate	Organic solvent coating (subcoating) Aqueous solvent coating (outer coating)	Hydrophobic substance: HPMC-based Opadry I^®^ Hydrophilic substance: PVA-based Opadry AMB^®^	Film-coated core tablets maintained their appearance for 30 days in 60%RH. Visual observation of the chosen formulation before and after 3 months exhibited no change, compared with a deformed commercial capsule that was packaged in Zymax blister film.	[40]
**Organic solvent coating**				
Aspirin (Acetylsalicylic Acid)	Pan-coating	Shellac	Shellac-coated tablets had significantly lower water uptake rates at 12.2% than HPMC-coated tablets at 19.2% at 100%RH. The difference was less pronounced at 75%RH with uptake rates at 3.0% compared with HPMC-coated tablets at 4.2%. This shows that shellac had higher potential for moisture protection than HPMC especially at high RH. However, the difference between the observed stability of drug was not as pronounced. Much lower shellac coating levels were needed for similar moisture protection as HPMC-coated tablets.	[41]
Solid dispersion of glimepiride and poly(ester amide) hyperbranched polymer	Pan-coating	HPMC phthalate Tablet core mixed with lactose (filler) and magnesium stearate (lubricant)	After 24 h in 75%RH, the increase in weight of solid dispersion powder at 29.5% (deliquescent) was significantly larger compared with the tablet core at 3.0% (hygroscopic) and coated tablets at 1.7% (slightly hygroscopic).	[42]
Diclofenac	Fluid bed	Hydrophobic substance: Hydrogenated Rosin (HR) Plasticizer: Hydrophilic: Glycerol (GLY) Hydrophobic: Dibutyl sebacate (DBS)	Hydrophilic plasticizer GLY was incompatible with hydrophobic HR due to their opposing natures. Flexible films cannot be formed as they cannot blend uniformly, resulting in brittle non-uniform films. HR films plasticized with DBS gave extremely low rates of water vapor transmission rates (10^−5^ g cm/cm^2^/24 h), which was very low compared with shellac.	[43]
**Dry powder coating**				
Pyridostigmine Bromide	Direct compression	Hydroxypropyl methyl cellulose (HPMC) Hydrophobic excipient with core: Avicel pH 102	HPMC-coated drug improved the hygroscopicity of pure drug slightly but it remained hygroscopic, as it softened in 2–3 days under ambient condition in comparison to the pure drug which transformed from solid to liquid state in 10 min. To improve hygroscopicity, water-insoluble excipient (Avicel pH 102) was added into the HPMC coating. The tablets did not show significant increase in moisture absorption up to 2 weeks at all conditions at 25 °C/60%RH, 30 °C/65%RH and 40 °C/75%RH,	[44]
Sennae fructus tablets	Dry powdered lipids coating: Hot-melt coating Coating of enteric coating: Fluid bed	Lipids: Medium chain triglyceride (MCT), Stearic acid (SA), Precirol^®^ ATO 5 (Pr), Compritol^®^ 888 ATO (Cp) Aq enteric coating: Eudragit^®^ L 30D-55 (EuL55)	The moisture permeability of the lipids follow this order: SA > MCT > Pr ~ Cp Only with Pr did the addition of EuL55 reduce moisture absorption. Other subcoatings of lipids with the addition of EuL55 showed no difference in their moisture absorption compared with just EuL55. The maximum reduction in hygroscopicity compared with tablet cores quantifies to 98% at 33%RH/RT, 96% at 43%RH/RT (Cp10 + EuL55 10) and 85% at 75%RH/RT (Pr10 + EuL55 10).	[45]
Fructose	One step dry-coated tablets (OSDRC) by compression	Crystallized compressed amorphous sucrose Plasticizer: Hydroxypropyl cellulose (HPC) and poly(vinylpyrrolidone) (PVP)	OSDRC tablets’ water vapor adsorption at 25 °C/75% in 15 h amount to a weight change of <1.0% in comparison to 5.5–6.0% in HPMC-coated tablets, showing its superior moisture-protective properties.	[46]

**Table 2:** Encapsulation by spray-drying using single or mixed polymers.

Drug/ Nutraceutical	Technique	Wall Material	Key Findings	Ref.
**Single polymer**				
Casein Hydrolysate	Spray-drying	Soy Protein Isolate (SPI)	Encapsulation increased hygroscopicity from 53 g/100 g to 106.99 g/100 g and 102.12 g/100 g, respectively, with SPI:hydrolysate ratios 70:30 and 80:20.	[57]
Casein Hydrolysate	Spray-drying	Maltodextrin: MD 10DE MD 20DE	Hygroscopicity of encapsulated hydrolysate was significantly less than free hydrolysate, improving from 53 g/100 g to 13.93 g/100 g (10DE) and 13.13 g/100 g (20DE).	[58]
Casein hydrolysates	Spray-drying	Maltodextrin (MD)	The microencapsulation process by MD (60% MD) significantly reduced values of hygroscopicity.	[59]
Flaxseed protein hydrolysates	Spray-drying	Maltodextrin (MD)	Hygroscopicity of encapsulated hydrolysate was significantly less than free hydrolysate, improving from from 39.2% to 17.4% in powders produced with carrier ratio 3:1.	[60]
Chicken meat protein hydrolysate	Spray-drying	Maltodextrin (MD) Gum Arabic (GA)	Hygroscopicity of encapsulated hydrolysate was significantly less than free hydrolysate, improving from 40.9 g/100 g to 15.9 g/100 g with 30% MD and 21.2 g/100 g with 30% GA.	[61]
Amaranthus Betacyanin Pigments	Spray-drying	Maltodextrin: MD 10DE MD 15DE MD 20DE MD 25DE Starches: Corn starch Modified corn starch	Hygroscopic properties increased with decreasing M_w_ of the wall materials. Hygroscopicity improved from 44.6–46.0 g/100 g to 40.9 g/100 g at the lowest DE investigated (i.e., 10DE MD). Corn starch gave the lowest hygroscopicity at 24.6 g/100 g.	[62]
Cactus pear juice	Spray-drying	Maltodextrin: MD 10DE MD 20DE	Hygroscopicity varied from 36.30–48.93 g/100 g. The least hygroscopic powders were obtained at the highest MD concentrations and pressure.	[63]
Acai (Euterpe Oleraceae Mart.)	Spray-drying	Maltodextrin: MD 10DE	The lowest hygroscopicity values were obtained when the highest MD concentrations used. Lower inlet air temperature and higher feed flow led to lower hygroscopicity.	[64]
Jujube powder	Spray-drying	Maltodextrin (MD)	Hygroscopicity was significantly affected by weight ratio of MD and feed flow rate (FFR). Lower hygroscopicities were obtained at higher MD and FFR. The highest MD concentration resulted in the least hygroscopicity. The optimum formulation had a hygroscopicity of 18.59%.	[65]
API-ILs: 1. -butyl-3-methylimidazolium Ibuprofenate (BMIm Ibu) 1. -butyl-3-methyl imidazolium Warfarinate (BMIm War) Choline Ibuprofenate (Cho Ibu) Choline Warfarinate (Cho War) Propranolol Saccharin (Pro Sac)	Spray-drying	Ethyl Cellulose: EC4 EC10 Maltodextrin: MD 6D	The physical stabilities of API-ILs encapsulated were tested by storing them at 25 °C/50%RH for up to 14 days. API-ILs encapsulated by MD were found to rapidly absorb water and transform from fine powder into extremely viscous sticky treacle-like liquid. In contrast, API-ILs encapsulated by EC remained as fine white powder and appeared unchanged upon storage.	[66]
**Mixed polymers**				
Whey protein hydrolysate	Spray-drying	Maltodextrin and β-cyclodextrin (MD and β-CD) MD	Hygroscopicity of encapsulated hydrolysate was significantly less than free hydrolysate, improving from 64.31 g/100 g to 43.09 g/100 g for MD-encapsulated and 36.99 g/100 g for MD/β-CD (1:1 *w*/*w*) encapsulated hydrolysate (70% wall).	[67]
Casein hydrolysate	Spray-drying	Gelatin and Soy Protein Isolate (GE and SPI)	All formulations were less hygroscopic than free hydrolysate, with the lowest at 27.23 g/100 g (20% hydrolysate, 80% carrier, (wall materials: 40% GE, 60% SPI)) compared to 53 g/100 g for free hydrolysate. Variations in GE and SPI concentration did not cause significant differences in hygroscopicity.	[68]
Purple sweet potato	Spray-drying	Maltodextrin (MD) and α-amylase	Hygroscopic moisture of spray dried flours treated with MD was at 2.9–3.0 g/kg, exhibiting lower hygroscopicity than control at 3.3 g/kg. Hygroscopicity increased at higher levels of amylase.	[69]
Whey protein hydrolysate	Spray-drying Or Freeze-drying	Whey Protein Concentrate and Sodium Alginate (WPC and ALG) WPC	Hygroscopicity of encapsulated hydrolysate obtained via both methods were significantly less than free hydrolysate. For spray-drying, the hygroscopic moisture of free hydrolysate at 26.69 g/100 g was reduced to 20.31 g/100 g (WPC) and 20.93 g/100 g (WPC/ALG). For freeze-drying, the hygroscopic moisture of free hydrolysate at 31.09 g/100 g was reduced to 26.28 g/100 g (WPC) and 24.40 g/100 g (WPC/ALG).	[70]
Soybean hydrolysates	Spray-drying Or Freeze-drying	Soy Protein Isolate and Maltodextrin (SPI and MD)	Hygroscopicity of encapsulated hydrolysate obtained via both methods were significantly less than free hydrolysate. For spray-drying, the hygroscopic moisture of free hydrolysate at 39 g/100 g was reduced to 18–20 g/100 g. For freeze-drying, the hygroscopic moisture of free hydrolysate at 41 g/100 g was reduced to 27–28 g/100 g.	[71]

**Table 3:** Encapsulation of bioactives by coacervation to reduce hygroscopicity.

Drug/ Nutraceutical	Technique	Wall Material	Key Findings	Ref.
Capsanthin	Complex coacervation Freeze drying	Soybean Protein Isolate/Chitosan (SPI/CS)	Microencapsulated capsanthin had enhanced stability against low and medium RH where it had improved retention rates from 42.77%, 54.37%, and 56.69% to 81.01%, 80.71% and 73.79% in RH 33%, 58%, 68%, respectively. It was less effective in protecting capsanthin in high humidity of 98%RH where its retention rate worsened from 68.99% to 45.81%.	[73]
Polyphenols from grape Juice Extract (GJE)	Extrusion into solution for complex coacervation Freeze drying	Gelatin/i-Carageenan (GE/i-Car)	Water uptake capacity (WU) is directly related to hygroscopicity. Blends containing higher amounts of GE had slightly lower WU. The lowest WU was observed at GE/i-Car at ratio 85:15, lower than that of 100% GE. WU between 8–12% was lower than WU of GJE at 32% after 100 h, showing how the freeze dried matrices can reduce the hygroscopicity of GJE.	[74]
Aspartame	Double emulsion complex coacervation Freeze drying	Gelatin/Gum Arabic (GE/GA)	Hygroscopicity were in the range of 10.73–13.43 g/100 g for all formulations, with no significant difference from free aspartame at 10.86 g/100 g.	[75]
Anthocyanin	Double emulsion complex coacervation Freeze drying	Gelatin/Gum Arabic (GE/GA)	Hygroscopicity ranged from 36–49 g/100 g, which was significantly less than anthocyanin at 85.22 g/100 g.	[76]
Anthocyanin	Double emulsion complex coacervation Freeze drying	Gelatin/Gum Arabic (GE/GA)	Hygroscopicity ranged from 37.05–49.05 g/100 g, which was significantly less than anthocyanin at 94.60 g/100 g.	[77]
Anthocyanin and tea extracts	Double emulsion complex coacervation Freeze dried extracts (FDE) Or Spray dried extracts (SDE)	Gelatin/Acacia Gum (GE/AG) Chitosan/Carboxymethylcellulose (CS/CMC)	Moisture content of SDE microcapsules at 2.39% and 3.23% were significantly lower than that of FDE at 4.88% and 4.91%, for GE/AG and CS/CMC-encapsulated capsules, respectively. GE/AG capsules had lower moisture content than CS/CMC capsules as CS/CMC layer was thicker, but their hygroscopicity difference was not large. SDE capsules had lower hygroscopicity at 21.4% and 21.8% than FDE microcapsules at 45.2% and 43.5%, for GE/AG and CS/CMC-encapsulated capsules, respectively.	[78]
Casein Hydrolysate	Double emulsion complex coacervation Freeze drying	Soybean Protein Isolate/Pectin (SPI/Pectin)	Hygroscopicity of free hydrolysate at 53 g/100 g was almost two times higher than the encapsulated samples at 20.08–24.38 g/100 g. The lowest value was obtained at the lowest hydrolysate content (50%). The more the content, the more hygroscopic the sample.	[72]
Mildronate	Double emulsion complex coacervation Dried at ambient temperature	Biodegradable: Poly(lactic acid) (PLA) Or Non-biodegrable: Polysterene (PS)	The polymer coatings decreased mildronate’s hygroscopicity in the long run (75%RH at 168 h) by more than two times, from 66.28%, to 26.18% in PLA and 22.04% in PS.	[79]
Probiotic Lactobacilli	Emulsification and external gelation/crosslinking Freeze drying	Alginate (ALG) Shellac (LAC) Whey Protein Isolate (WPI) All formulations have sucrose: ALG ALG/LAC ALG/WPI ALG/WPI/LAC	The order of hygroscopicity is as follows ALG/WPI > ALG > ALG/WPI/LAC > ALG/LAC based on moisture absorption isotherms and vapor absorption rates of the microcapsules. LAC addition had significantly reduced hygroscopicity, which can be seen in the storage stability afforded by the encapsulation, in the otherwise quick inactivation of probiotics.	[80]

**Table 4:** Co-processing with excipients to reduce hygroscopicity.

Drug/ Nutraceutical	Technique	Excipients	Key Findings	Ref.
Nitrofurantoin anhydrate	Wet granulation	Amorphous: Low-substituted Hydroxypropyl cellulose (HPC) Pregelatinized starch Partially amorphous: Silicified Microcrystalline cellulose (MCC) Crystalline: α-lactose monohydrate	Only the amorphous excipients impeded hydrate formation of the drug at high water content. The hygroscopic partially amorphous excipient hindered hydrate formation of drug at low water contents. The crystalline excipient was unable to control hydrate formation of drug.	[82]
Ginsenoside from Red Ginseng Extract	Dissolution and freeze drying Mortar	Silicon Dioxide (SiO_2_)	The water sorption rate of the extract was at <20% from 30%RH to 70%RH, compared to solid dispersion with SiO_2_ at <12%. Visual observation of the solid dispersion yielded no observable differences before and after storage in various humid conditions, from 30% to 70%RH for 25 days at 30 °C. The desorption isotherms show that the dispersion had negligible hysteresis, which showed the reversible change in water content and the non-hygroscopic nature of the powder.	[83]
Aspirin (Acetylsalicylic Acid)	Physical mixture	Highest to lowest crystallinity: Cladophora cellulose Microcrystalline cellulose (MCC-SLM) Agglomerated micronized cellulose (AMC) Low crystallinity cellulose (LCC)	The lowest degradation rates were found in formulations consisted of the lowest crystallinity LCC despite its highest moisture.	[84]
Aspirin (Acetylsalicylic Acid)	Physical mixture	High crystallinity cellulose (HCC) Microcrystalline cellulose (MCC) Low crystallinity cellulose (LCC) Lactose	Drug degradation increased with high amounts of crystallinity cellulose HCC and MCC. It was lowest and decreased with higher amount of LCC despite its higher moisture content.	[85]
Traditional Chinese Medicines (TCM)	Powder: Wet granulation Oven drying Tablets formation: Compression	Porous calcium silicate (Florite RE, FLR)	Wet granulation was a success with the addition of FLR. It is suitable for use in wet granulation of very hygroscopic powders as water retained in FLR will be transferred to the added hygroscopic material very slowly, resulting in granules.	[86]
Limaprost	Dissolution and freeze drying	Dextran40 Dextrin Pullulan	Although water content after storage was extremely high at >10%, the drug was stabilized.	[87]
Herbs: Radix ophiopogonis Rhizoma polygonati	Physical mixture Oven drying	Dextrans T10, T40, T70	The moisture sorption was depressed by increasing mass of dextrans. Dextran also increased the T_g_ of the extracts at all RH, reducing their tackiness and ability to absorb water.	[88]
Physalis peruviana fruit extract	Wet granulation Oven drying Or Fluidized bed drying	Combination of corn starch and Microcrystalline cellulose (MCC)	The formulation improved the hygroscopicity of powder slightly, changing it from class III (moderately hygroscopic) to class II (slightly hygroscopic) powder. It also helped to avoid deliquescence of the extract.	[89]
Raw date	Physical mixture Oven drying Hammer mill	Maltodextrin (MD)	MD increased Tg The higher the MD content, the lower the hygroscopicity. Hygroscopicity value was 6.2% at MD to date ratio of 35:65, and 4.0% at 50:50. MD:date at 50:50 remained free flowing even after a year of storage.	[90]
Betahistine dihydrochloride	Direct compression	Quick Tab™ (mix of tricalcium phosphate, microcrystalline cellulose, povidone, cross povidone)	After 3 months in 25 °C/60%RH accelerated stability testing conditions, the formulation appearance was found to be satisfactory with no change. Its weight variation was also insignificant.	[91]
Betahistine (BTH)	Kneading and oven drying Or Dissolution and freeze drying	Inclusion complex: β-cyclodextrins (β-CD)	Compared with BTH which liquefied completely after 100 min, the solids obtained from both kneading and freeze drying were not completely liquefied even after a month. At 300 h in 75%RH, BTH’s weight varied by 70–80%, compared with that of the solids obtained by both methods which increased by <10%. At 240 min in 33%RH, BTH completely liquefied, compared with the weight variation in the solids obtained by both methods which increased by <10%.	[92]

**Table 5:** Co-crystallization as a method to reduce hygroscopicity.

Drug/ Nutraceutical	Technique	Co-Former	Key Findings	Ref.
Phloroglucinol (SPF)	Solvent evaporation	Progesterone (Prog)	In dynamic vapor sorption, SPF absorbed 25% water to convert to dihydrate at 40%RH. Prog-SPF and Prog-SPF-HH (hemihydrate) had remarkable hygroscopic advantage over SPF, as they absorbed 0.5% and 0.3% water, respectively, at 80%RH. Furthermore, Prog-SPF absorbed 0.3% and 0.4% water at 60% and 80%RH in 2 weeks, showing their non-hygroscopic nature.	[94]
Berberine Chloride (BCl)	Solvent evaporation	Succinic acid (SUA) Glutaric acid (GLA) Adipic acid (ADA) Pimelic acid (PIA)	In dynamic vapor sorption, BCl absorbed 8.1–10% water to convert to dihydrate from 10% to 70%RH, and absorbed 11– 16% water to convert to tetrahydrate from 80% to 90%RH. All cocrystals adsorbed negligible amounts of moisture of <1.2% up to 70%RH. Hygroscopicity follows the ascending order of BCI-SUA < BCl-GLA < BCl-ADA < BCl-PIA < BCl.2H_2_O. BCl-SUA and BCI-GLA were less hygroscopic than the other 2 cocrystals as they did not form hydrates when exposed to high RH.	[95]
Isosorbide (ISO)	Solvent evaporation	Piperazine (PZ) Hydrochlorothiazide (HCT) 3,5-dihydroxybenzoic acid (35DHBA) Gallic acid (GaA)	The critical RH of cocrystals at 56–85%RH were higher than that of of ISO at 48%RH. At 95%RH, ISO and ISO-PZ deliquesced, whereas ISO-HCT, ISO-35DHBA and ISO-GA remained solid.	[96]
Palmatine Chloride (PMTCl)	Solvent evaporation	Gallic acid (GaA)	The hygroscopic stability of PMTCl-GA salt cocrystals were significantly better than PMTCl. At 75%, 80% and 95%RH, PMTCl took more days to reach moisture equilibrium to gain 7.07%, 16.46% and 19.01% water mass at each RH, respectively, as compared to 2.83%, 4.84% and 5.78% moisture gained by PMTCl-GA.	[97]
Metformin (MET)	Solvent evaporation	Epalrestat (EP)	The hygroscopicity of MET and METCl (commercial) were higher than EP-MET and EP-MET monohydrate (MH). At 95%RH, the weight of MET increased 63%, EP-MET increased 0.25%, EP-MET MH increased <1%, and METCl increased 1.54%.	[98]
Sodium Valproate (VAL)	Solvent evaporation &and Liquid-assisted grinding	Carbamazepine (CBM) Tromethamine (TMA)	TMA-VAL absorbed almost no water at <1% up to 75%RH after 1 week, whereas sodium VAL absorbed 70.02%. CBM-VAL absorbed <10% at 100%RH after 1 week, whereas sodium VAL absorbed 144.69%.	[99]
Amorphous vemurafenib (VEM)	Liquid-assisted grinding and Solvent evaporation	D-camphorsulfonic acid (D-CSA) L-camphorsulfonic acid (L-CSA) DL-camphorsulfonic acid (DL-CSA)	Improved hygroscopicity was observed as amorphous VEM absorbed 2.45% water at 95%RH, and cocrystals VEM-D-CSA, VEM-L-CSA, and VEM-DL-CSA absorbed 1.05%, 1.10% and 1.17%, respectively.	[100]
Berberine Chloride (BCl)	Liquid-assisted grinding	L-Lactic acid (LA)	In dynamic vapor sorption, BCl rapidly absorbed water at 8.8% to convert to dihydrate from 0% to 10%RH, remained stable up to 70%RH, and absorbed significant amount of water from 70% to 90%RH to convert to tetrahydrate. BCl-LA gained insignificant moisture at 0.3% from 0 to 10%RH, remained stable up to 70%RH, and absorbed 2 water molecules to become BCl-LA.H_2_O from 70% to 95%RH. The cocrystal exhibited lower hygroscopicity than BCl.	[101]
Berberine Chloride (BCl)	Liquid-assisted grinding	Citric acid (CA)	In dynamic vapor sorption, BCl rapidly absorbed water to convert to dihydrate from 0% to 10%RH, gained weight gradually via surface water adsorption to 70%RH, and absorbed significant amount of water to become tetrahydrate from 70% to 90%RH. BCl-CA gained insignificant moisture at < 2% from 0 to 70%RH, gained moisture gradually at 8% from 70% to 95%RH. The lack of significant hysteresis and step weight gain from 70% to 95%RH showed that BCl-CA did not transform to a hydrate at 95%RH, and that it exhibited greater physical stability over BCl.	[102]
Levofloxacin (LVFX)	Neat grinding	Metacetamol (AMAP)	In dynamic vapor sorption, LVFX absorbed water at 2.2% to become a hydrate when RH increased from 0% to 10%. LVFX-AMAP absorbed water at 0.3% at 95%RH, indicating its non-hygroscopic properties.	[103]
L-Lactic acid (LA)	Melt crystallization	D-tryptophan (D-T) 3-nitrobenzamide (3-N)	In dynamic vapor sorption, LA deliquesced and had a net increase of 1.3157 g/g sample between 0% to 90%RH, compared to the little mass increase in cocrystals at 0.0017 g/g sample of LA-D-T and 0.0299 g/g sample for LA-3-N. Visual observation at RH96% confirmed the deliquescence of LA, and how both cocrystals remained the same.	[104]

## Data Availability

Not applicable.

## Abbreviations

**Abbreviation**	**Definition**
β-CD	β-cyclodextrin
ALG	Alginate
AMAP	Metacetamol
ANC	Anthocyanin
API-IL	Active Pharmaceutical Ingredient—Ionic Liquid
AS	Aspartame
BCl	Berberine Chloride
CA	Citric Acid
CMC	Carboxymethylcellulose
CS	Chitosan
EC	Ethyl Cellulose
EP	Epalrestat
FDA	Food and Drug Administration
FLR	Calcium Silicate
GA	Gum Arabic
GaA	Gallic Acid
GE	Gelatin
GRAS	Generally Recognised As Safe
HPC	Hydroxypropyl Cellulose
HPMC	Hydroxypropyl Methyl Cellulose
i-Car	i-Carrageenan
L-HPC	Low-substituted Hydroxypropylcellulose
LAC	Shellac
LCC	Low Crystallinity Cellulose
LVFX	Levofloxacin
MD	Maltodextrin
MET	Metformin
METCl	Metformin Chloride
M_w_	Molecular Weight
OSDRC	One-step dry-coated
PLA	Poly(lactic acid)
PMMA	Poly(methacrylate-methylmethacrylate)
PS	Polystyrene
PSAA	Polymeric Surface-active Agent
PVA	Polyvinyl Alcohol
PVP	Poly(vinylpyrrolidone)
RH	Relative Humidity
SA	Stearic Acid
SPI	Soy Protein Isolate
TCM	Traditional Chinese Medicine
T_g_	Glass transition temperature
THEDES	Therapeutic Deep Eutectic Solvents
VEM	Vemurafenib
W/O	Water/Oil
W/O/W	Water/Oil/Water
WPC	Whey Protein Concentrate
WPI	Whey Protein Isolate
